# Diagnostic Accuracy of a New Antigen Test for SARS-CoV-2 Detection

**DOI:** 10.3390/ijerph18126310

**Published:** 2021-06-10

**Authors:** Marina Di Domenico, Alfredo De Rosa, Francesca Di Gaudio, Pietro Internicola, Cinzia Bettini, Nicola Salzano, Davide Castrianni, Andrea Marotta, Mariarosaria Boccellino

**Affiliations:** 1 Department of Precision Medicine, University of Campania “Luigi Vanvitelli”, 80138 Naples, Italy; marina.didomenico@unicampania.it; 2 Department of Biology, College of Science and Technology, Temple University, Philadelphia, PA 19122, USA; 3 Multidisciplinary Department of Medical-Surgical and Dental Specialties, University of Campania “Luigi Vanvitelli”, 80138 Naples, Italy; alfredo.derosa@unicampania.it (A.D.R.); andrea.massimo.62@gmail.com (A.M.); 4 Department of Health of the Region of Sicily, CRQ (Regional Laboratory Quality Control Centre), 90145 Palermo, Italy; francescadigaudio@unipa.it; 5 Anesthesia and Reanimation Service “Santa Maria della Pietà” Hospital, 80026 Casoria, Italy; pietrointernicola@gmail.com; 6 Laboratory and Clinical Analysis Service “Santa Maria della Pietà” Hospital, 80026 Casoria, Italy; cinzia.bettini@gmail.com; 7 Italian Ministry of Health, USMAF SASN, 80133 Naples, Italy; salzano.nicola@gmail.com (N.S.); davide.castrianni@gmail.com (D.C.)

**Keywords:** respiratory disease COVID-19, SARS-CoV-2, ELISA, cyto-salivary test, RT-PCR, lateral flow test

## Abstract

Background and aims: Quick and reliable diagnostic tools play an important role in controlling the spread of the SARS-Cov-2 pandemic. The aim of this study was to evaluate the diagnostic accuracy of a new cyto-salivary antigen test aimed at detecting the presence of antigens for SARS-CoV-2, as compared by the gold standard RT-PCR and a lateral flow test. Methods: A total of 433 healthy volunteers were enrolled in the study and the sensitivity and specificity of the new cyto-salivary antigen test were calculated, as compared to the RT-PCR nasopharyngeal swab and to the lateral flow test. Results: A total of 433 samples were collected and tested at the Mediterranean Fair in Palermo from February 2021 until April 2021. The new cyto-salivary antigen had a sensitivity of 100% and a specificity of 94.2%. The sensitivity and the specificity of the lateral flow test were 55% and 100%, respectively. Conclusions: The new cyto-salivary antigen test detected more positive cases than the RT-PCR in a sample of asymptomatic subjects, demonstrating to be a promising tool for a more sensitive diagnosis of COVID-19. Further studies are warranted to better characterize its diagnostic accuracy.

## 1. Introduction

The new coronavirus pandemic, Coronavirus Disease 2019 (COVID-19), has spread from Wuhan, China, to all countries of the world. Coronaviruses (CoV) are a family of related microorganisms that infect the respiratory tract in humans. Seven genera are known of which four, including human coronavirus 229E, human coronavirus OC43, human coronavirus NL63 and human coronavirus HKU1, cause relatively mild respiratory symptoms [1,2]. The others, Severe Acute Respiratory Syndrome Coronavirus (SARS-CoV), Middle East Respiratory Syndrome Coronavirus (MERS-CoV), and Sever Acute Respiratory Syndrome Coronavirus 2 (SARS-CoV-2) lead to severe respiratory diseases and clinical fatal prognosis. Worldometer COVID-19 data showed approximately 149.4 million confirmed cases, 3.2 million deaths and 127.1 million recovered patients, on 28 April 2021. Since its inception, the COVID-19 emergency has profoundly marked medicine and science, bringing significant damage and challenges around the world.

COVID-19 shows a biphasic pattern of disease consisting of an early viral response phase and a second inflammatory phase. The symptoms reported by the first infected patients were: fever, cough, muscle aches and fatigue. Less frequent symptoms included cough with sputum or hemoptysis, headache and diarrhea, and some had shortness of breath. Furthermore, computed tomography examination revealed the presence of pneumonia in infected people [3]. However, the symptoms of SARS-CoV-2 infection are not specific, and the presentation of the disease can range from no symptoms (asymptomatic subject) to severe pneumonia and death. Most people with asymptomatic infections are unaware of their infection status but are still contagious to others as the initial viral load can be high [4]. This further increases the difficulty of current SARS-CoV-2 infection prevention and control efforts. Usually, SARS-CoV-2 infection almost always starts deceptively as if it were a common flu syndrome with symptoms such as those of the common cold or simple pharyngitis. The duration of these symptoms is variable because it depends on the immune strength of the affected individual [5]. Del Rio C et al. showed that long-term pulmonary, cardiological, and neurological complications have also been reported in COVID-19 cases [6]. SARS-CoV-2 is less fatal but much more transmissible with an estimated basic reproductive rate (R_0_) of 2.5 (range 1.8–3.6) compared to 2.0–3.0 for SARS-CoV and 0.9 MERS-CoV [7].

Viral infections are difficult to cure and require robust host defense mechanisms to fight them; however, early diagnosis and surveillance of the disease can block its spread.

Carrying out rapid screening tests for COVID-19 is of fundamental importance to prevent and control infections. The development of new diagnostic methods for SARS-Cov-2 infection that are rapid, accurate, sensitive, and inexpensive is extremely important for surveillance, infection control, and clinical management of the disease. Reverse-transcription polymerase chain reaction (RT-PCR) is currently the gold standard for the diagnosis of SARS-CoV-2 infection [8]. However, this technique requires the use of well-equipped laboratories with sophisticated equipment and highly qualified personnel, making it unsuitable for rapid diagnosis directly in the field and for mass screening programs. In addition, another problem for RT-PCR assays is the high rate of false negatives (30% to 50%) [9]. The WHO director-general in the opening speech at the media briefing on COVID-19 in March 2020 had already highlighted that isolation of infected individuals was necessary to fight the pandemic, in fact, he urged all countries to test, test, test [10]. However, few countries have managed to increase testing capacity to gather data needed to facilitate public health decisions regarding return to work, school reopening, sporting events and travel, and to allow for the loosening of restrictions. All significant consequences for the economy and public health. Antigen tests diagnose active COVID-19 infection by detecting specific SARS-CoV-2 viral proteins in various types of samples. They can be performed outside the laboratory and are available as single-use, lateral flow, antigen detection rapid diagnostic tests that can be read visually or using a small handheld device.

Rapid antigen tests can be performed onsite in mass testing, are inexpensive compared to RT-PCR, do not require specific and expensive machinery and the results are available within 15–30 min [4], which could serve to evaluate chains of infection and their interruption. Recently, Dinnes J et al. performed a meta-analysis which revealed that the average sensitivity and specificity of the rapid antigen tests for SARS- CoV-2 was 56.2% and 99.5%, respectively [11], showing that the main limitation of such tests is the low sensitivity.

In Central Italy, a low prevalence of SARS-Cov-2 infection was found in Health Workers although higher than the rest of the population. The serological IgM test does not appear useful for the diagnosis of SARS-CoV-2 while the IgG serological test showed a higher diagnostic performance when executed at least two weeks after the RT-PCR test [12].

Recently a new qualitative, rapid, sensitive, non-invasive, and specific method for the diagnosis of SARS-CoV-2 infection which is based on the recognition of specific antigens of the virus was proposed [13]. It is an ELISA technique that can be performed in environments outside the laboratory with immediate chemocolorimetric response. The method is used in a kit which is easily transportable and usable in the field “patient side” as it does not require special equipment. In particular, such kit allows the determination of two SARS-CoV-2 proteins, the S protein (Spike protein) and the N protein (Nucleocapsid) through a double use of primary antibodies to unequivocally guarantee the specificity and signal sensitivity. The kit uses reagents stable at room temperature and allows to qualitatively identify the presence of antigens characterizing the COVID-19 coronavirus infection by cyto-salivary sampling. The search for antigens allows to identify the incubation phase or the first phases of the infection where the antibody response is still low and the characterizing symptoms not yet evident.

The aim of this study was to perform a clinical evaluation of a new cyto-salivary antigen test for SARS-CoV-2 diagnosis, in asymptomatic infected persons, compared to the RT-PCR and a commonly used lateral flow test for the detection of SARS-CoV-2. The kit used in the study has a REF NUMBER: COV2PC19AL and the production LOT #: CO-0006Z12.

## 2. Materials and Methods

### 2.1. Patients and Samples

We collected a total of 433 cyto-salivary specimens from healthy individuals from February 2021 until April 2021 at Military Checkpoint during the Mediterranean Fair in Palermo. All subjects (median age—37 years, range—15–78) were healthy volunteers attending the Fair. The enrolled people accepted to be included in the study, and to be tested once with 3 diagnostic tests for SARS-COV-2 detection: the standard RT-PCR, the new cyto-salivary antigen test and the lateral flow test. Then, we analyzed the diagnostic accuracy of the new test based on an enzyme-linked immunosorbent assay (ELISA) and the lateral flow test vs the RT-PCR. The swab, execution, and evaluation of the test were performed by personnel specifically enrolled by the suppliers of the kits and by the personnel enrolled by the structures involved in the study according to the procedures indicated by the methods. The procedures were identical for all subjects. Precisely, each volunteer was seated and, without any mouth rinsing, was subjected to the first biological sampling for the lateral flow test, and immediately in sequence the collection for the cyto-salivary test was carried out. Subsequently, the subject was accompanied to next location for the execution of the swab for the RT-PCR test.

The study was conducted according to the guidelines of the Declaration of Helsinki, and approved by CRQ-Sicilian Region, Department of Health, Regional Department for Strategic Planning, Department for Health Activities and Epidemiological Observatory with Prot./CRQ/n. 864. Written informed consent was obtained from each subject prior to the initiation of any study-related screening procedure.

### 2.2. Inclusion and Exclusion Criteria

#### 2.2.1. Inclusion Criteria

The volunteers accepted to sign informed consent to be included in the cohort enrolled for screening, and agreed to undergo three samples for the three different assays.

#### 2.2.2. Exclusion Criteria

Subjects who refused to sign informed consent or who were unable to sign it for inability to understand and wanted to limit the study.

### 2.3. Data Collection

Clinical data were collected for each subject upon presentation of a questionnaire including any contact with people affected by COVID-19 or with people from areas at risk, the presence in the previous 14 days of symptoms such as fever, cough, asthenia, headache, vomit and/or diarrhea, disturbances in smell and/or taste, and comorbidities. Comorbidities included in the questionnaire were hypertension, cardiovascular disease, chronic lung disease, diabetes, chronic renal failure, active cancer including lymphoproliferative disease, severe immunosuppression, immunosuppressive therapy, pregnancy, and obesity.

### 2.4. Cyto-Salivary Antigen Test

We used a newly developed SARS-CoV-2 cyto-salivary antigen test system (Portable COVID-19 Antigen Lab Test by Stark, REF NUMBER: COV2PC19AL and the production LOT #: CO-0006Z12) [13]. The method is based on ELISA technique with a chemocolorimetric result on a membrane adsorbed with antibodies for the specific virus antigens (Spike protein and Nucleocapsid), as reported [13]. Briefly, the cyto-salivary sampling collected in a non-invasive way with the brushing technique on the mucous membrane of the oral cavity and on the back of the tongue is processed at room temperature following precise steps starting from the extraction of viral proteins. The antigens are then intercepted by a double sandwich of polyclonal and monoclonal SARS-CoV/SARS-CoV-2 (COVID-19) spike antibody and SARS-CoV/SARS-CoV-2 (COVID-19) nucleocapsid antibody forming immune complexes which, conjugated to enzymatic detector systems, activate a positive colorimetric reaction to the virus. The application of monoclonal antibodies is crucial for the establishment of a technique with high sensitivity and specificity. The test result is qualitative: negative or positive, or null if it was performed incorrectly. The result of the antigen–antibody reaction can be directly visible after about 30 min, to the naked eye without analytical instrumentation. The diagnostic efficacy is characterized by an analytical sensitivity (LOD) equal to 4.2 × 10^−8^ mol/L for Nucleocapsid, and 1.5 × 10^−8^ mol/L for Spike protein. The kit box is organized in rows of nine wells/stations containing buffers, lysis systems, detection systems stable at room temperature and dried antibodies stable at room temperature. The kit is equipped with tools for the collection of biological material and a support for the PVDF strip that displays the results of the test following the instructions given.

### 2.5. Lateral Flow Test

The diagnostic comparison between the new cyto-salivary antigen test and a common lateral flow test was processed on-site using the Panbio™ COVID-19 Ag Rapid Test Device (Abbott Diagnostic GmbH, Jena, Germany) for the rapid and qualitative detection of the SARS-CoV-2 virus. It is a lateral-flow-format test that uses immunochromatography with colloidal gold, and it is designed to be performed at the patient care site by trained healthcare personnel. The nasopharyngeal swab was used for specimen collection and the results were interpreted within 15–20 min following the manufacturer’s instructions. This kit detects the presence of a specific virus protein, the nucleocapsid (N) protein (the target protein of all point-of-care antigen tests), on a membrane-based assay. The sample from the swab is mixed with approximately 300 μL of buffer, and then 5 drops are dispensed into the device [14,15].

### 2.6. RT-PCR Testing

RT-PCR assay for SARS-CoV-2 (QuantStudioTM, Thermo Fisher Scientific, Waltham, MA, USA) using nasopharyngeal swabs (NPS) in 3 mL viral transport medium (VTM) was assessed [16,17]. RNA from all samples was extracted using MagMAX Viral/Pathogen II (MVP II) Nucleic Acid Isolation Kit (Thermo Fisher Scientific, Waltham, MA, USA) following the instructions recommended by the manufacturer. To calculate the viral loads (VL) as SARS-CoV-2 genome copy numbers per mL, a standard curve was obtained by using a quantified supernatant from a cell culture isolate of SARS-CoV-2. The target genes of the RT-PCR were Orf-1ab, N Protein and S Protein. Samples with a cycle threshold (Ct) value from 10 to 35 were considered positive, higher values were considered negative. Samples with Ct equal to or greater than 35 were repeated at least twice being the viral load rather low.

### 2.7. Statistics

We estimated that 430 subjects were needed to have a 90% power to detect sensitivity and a specificity of the new cyto-salivary antigen test of at least 80%, assuming a prevalence of positive RT-PCR of 6.5%. The proportion of positive cases among the tested cases in the period when the study was conducted was fluctuating between 8% and 10% (Data from: Protezione Civile, visible at: https://lab24.ilsole24ore.com, accessed on 27 May 2021). Since the study was conducted in healthy subjects with no indication for an RT-PCR, we reduced the lower limit of this expected proportion (8%) of 20%, setting this expected proportion to 6.5%. Assuming a loss of 10% of subjects with a valid set of 3 tests, we planned to enroll 480 subjects into the study. We estimated the sensitivity and specificity of the cyto-salivary test against the molecular test (RT-PCR) and compared it to the sensitivity and specificity of the lateral flow test with 95% Confidence Intervals (CI) estimated using the variance of proportions. We run a chi-square test to assess whether sensitivity and specificity were significantly higher than 50%. MedCalc^®^ statistical software (Version 12.3, Mariakerke, Belgium) was used for analysis.

## 3. Results

From February 2021 until April 2021, screening was performed to evaluate the diagnostic accuracy of the new cyto-salivary antigen test and the lateral flow test vs the RT-PCR. For this purpose, we enrolled 497 subjects in the study. Of them, 64 were excluded since they did not receive all the three tests, and 433 subjects were included in the analysis.

**Figure 1:**
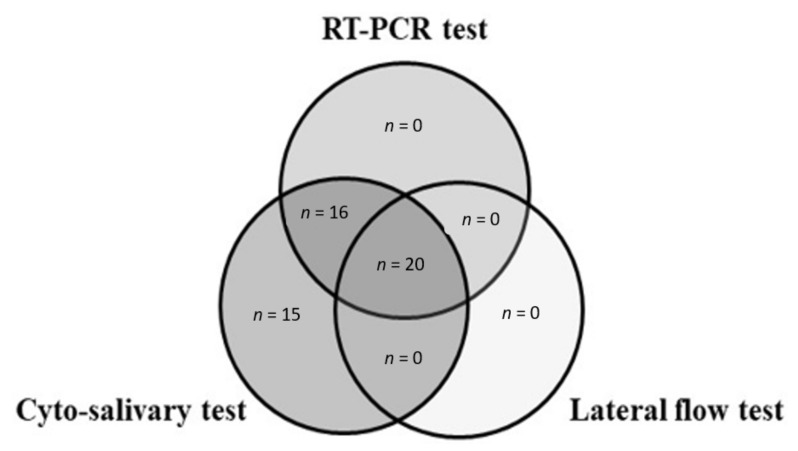
Venn plots show the overlapping of positive cases of the three tests performed (cyto-salivary, lateral flow and RT-PCR), *n* = number of positive cases.

Table 1 and Table 2 report the results about the new cyto-salivary test and the lateral flow test, respectively, vs the RT-PCR. The RT-PCR detected 36 (8.3%) positive cases and 397 (91.7%) negative cases. The cyto-salivary test detected the highest number of positive cases: Fifty-one (12%) were positive and 382 were negative (88%). The lateral flow test detected 20 (4.6%) positive cases and 413 (95.6%) negative cases. Table 3 reports the comparison of the lateral flow test and the cyto-salivary antigen test. The positive results detected by the three tests were represented by Venn diagrams (Figure 1). As shown in the figure, the Venn plots identified 20 positive cases common to the three tests performed (cyto-salivary, lateral flow and RT-PCR) and 16 positive cases common to cyto-salivary and RT-PCR tests.

Statistical analysis by using the MedCalc Statistical calculators method showed a sensitivity of the cyto-salivary test was 100% (95% CI = 90.3–100.0) and the specificity was 96.2% (95% CI = 93.8–97.9) (*p* < 0.001). The reported lateral flow test sensitivity was 56% (95% CI = 38.1–72.6%) and the specificity was 100% (95% CI = 99.1–100%) (*p* < 0.001). These results confirm the high sensitivity and specificity of the new cyto-salivary antigen test. 

## 4. Discussion

This study provides an estimate of the diagnostic accuracy of a new cyto-salivary antigenic test. The standard RT-PCR, the new cyto-salivary antigen test and the lateral flow test were performed on 433 healthy volunteers. The analysis of the results revealed high sensitivity and specificity (respectively 100% and 96.22%) of the new cyto-salivary antigen test. This is an important finding because some asymptomatic individuals with SARS-CoV-2 infections might have a considerable influence on the spreading of this virus. The cyto-salivary antigen test provided a qualitative, rapid, sensitive, and specific diagnosis of SARS-CoV-2 infection based on the recognition of virus-specific antigens (nucleocapsid and spike). This recognition, implemented through the dual use of primary antibodies, unequivocally guarantee the signal specificity and sensitivity [13]. Both saliva and epithelial cells from the mucous membrane of the oral cavity, into which the virus may have penetrated, are collected through cyto-salivary sampling with opportune cytobrush. These cells are suitably lysed, thus increasing the availability of viral proteins accentuating the sensitivity of the test. The high sensitivity of the test is also due to the use of a detector enzymatic system (Alkaline phosphatase) which performs its maximum activity at room temperature, allowing optimal signal amplification. The method is based on ELISA assay and is used in a kit which makes it easily transportable and usable in the field “patient side” as it does not need any particular equipment. The ELISA technique, aimed at recognizing antigens or antibodies, has been recognized since 1972 as a highly sensitive technique [18]. Moreover, the ELISA technique leads to adequate analytical specificity which depends on the affinity between antigen and antibody. Thanks to its high sensitivity, the new cyto-salivary antigenic test could allow to monitor the spread of the virus in the population almost in real time and to have a more accurate estimate of cases. It could prove very useful in ports and airports, schools and universities, workplaces and public offices to contain the transmission of the virus and prevent the need to resort to more restrictive measures or return to lockdown.

The test principle of most antigen tests is based on antibodies targeting the SARS-CoV-2 proteins, while the reference standard PCR is based on the amplification of RNA to millions of copies. In using rapid antigen tests, sensitivity and specificity are two essential components that provide information on their performance to accurately detect the presence of the virus in infected individuals. It was shown that the sensitivity of rapid antigen tests is expected to be lower; however, recent analysis has shown that in this context, sensitivity is less important than test frequency and turn-around time [19,20].

The WHO recommended for antigen tests minimum sensitivity as 80% and specificity as 97%, compared with a molecular test. Moreover, WHO recognizes that despite lower sensitivity than molecular tests, antigen tests offer the possibility of rapid, inexpensive detection of SARS-CoV-2 in individuals who have high viral loads and, hence, are at high risk of transmitting the infection to others [21,22].

Mass testing is the fulcrum of the strategy to contain the current SARS-CoV-2 pandemic. Screening strategies are especially recommended in exposed or high-risk Health Workers (HWs) [12]. A large number of tests makes the spread of the infection visible and allows infected people to be quickly extracted from the circulation. Thus, a rapid and accurate diagnosis is essential to allow not only the tracing of contacts, but also the administration of treatments appropriate to the severity of the disease. SARS-CoV-2 has been identified and sequenced by a Chinese team, allowing doctors around the world to perform RT-PCR on oropharyngeal or nasopharyngeal swabs in patients with suspected COVID-19 [23]. However, performance problems were encountered, particularly with regard to sensitivity [24]. In addition, a high false negative rate in RT-PCR tests for SARS-CoV-2 in hospitalized patients with a clinical diagnosis of COVID-19 is well documented in several studies [25,26]. Furthermore, the results of RT-PCR tests were variable and unstable when repeated multiple times on the same patient, probably due to insufficient sample or laboratory error or problems related to the transport of biological samples. Therefore, RT-PCR tests should not be considered as the only indicator for the diagnosis, treatment, and discharge of hospitalized patients with a clinical diagnosis of COVID-19. Therefore, the diagnosis for SARS-Cov-2 infection must be based not only on the results of the RT-PCR test but also on the results of other tests and on the clinical presentation of the patient. The strength of the present study is the high sensitivity of the method. We only examined one type of testing scenario based on asymptomatic enrolled subjects. Therefore, subsequent studies will be necessary by recruiting a greater number of patients and also evaluating the immunological aspect as well as the subsequent clinical evolution in asymptomatic subjects which are results positive to test. Additional rigorous studies, including symptomatic subjects at the time of interview, are needed to establish the optimal performance characteristics of the cyto-salivary antigen test.

## 5. Conclusions

The new assay for SARS-CoV-2 antigen detection showed high sensitivity and specificity as well as clinical efficiency (speed and ease of use). The high sensitivity of the new cyto-salivary antigenic test might allow an early diagnosis, even in those subjects with borderline SARS-CoV-2 positivity. Its signal cut-off point makes it unique among diagnostic systems. In addition, another strength of this test that is not present in other systems is that of simultaneously detecting two specific viral antigens, which certainly increases the specificity. Furthermore, the use of a cell lysis buffer in the first step of the kit allows for obtaining a greater viral load in solution, which accentuates the sensitivity. The results of this study show that the kit could be a valid tool for detecting SARS-CoV-2 infection, in particular it can be very useful in monitoring the incidence and progress of the disease, as well as evaluate its severity over time; moreover, it allows for operating a mass screening that can quantify the epidemic state of community environments, such as schools, airports, stations, and ports, preventing clusters or outbreaks. However, we consider these results as a clinical preliminary approach for Coronavirus Disease 2019 due to the absence of symptomatic patients enrolled for the screening; therefore, the recruitment of a greater number of subjects including symptomatic ones will allow us to reach more solid conclusions.

## Figures and Tables

**Table 1:** Results of the screening of the new cyto-salivary antigen test compared with RT-PCR in asymptomatic subjects for SARS-CoV-2 detection.

Subjects	RT-PCR Positive	RT-PCR Negative	Tot
Cyto-salivary positive	36 (100%)	15 (3.8%)	51
Cyto-salivary negative	0	382 (96.2%)	382
Tot	36	397	433

**Table 2:** Results of the screening of the lateral flow test compared with RT-PCR in asymptomatic subjects for SARS-CoV-2 detection.

Subjects	RT-PCR Positive	RT-PCR Negative	Tot
Lateral flow positive	20 (56%)	0	20
Lateral flow negative	16 (44%)	397 (100%)	413
Tot	36	397	433

**Table 3:** Results of the screening of the lateral flow test compared with cyto-salivary antigen test.

Subjects	Cyto-Salivary Positive	Cyto-Salivary Negative	Tot
Lateral flow positive	20	0	20
Lateral flow negative	31	382	413
Tot	51	382	433

## Data Availability

The data presented in this study are available on request from the corresponding author.
